# A Class I HDAC Inhibitor Rescues Synaptic Damage and Neuron Loss in APP-Transfected Cells and APP/PS1 Mice through the GRIP1/AMPA Pathway

**DOI:** 10.3390/molecules27134160

**Published:** 2022-06-29

**Authors:** Ying Han, Le Chen, Jingyun Liu, Jie Chen, Chunyang Wang, Yu Guo, Xuebin Yu, Chenghong Zhang, Haiying Chu, Haiying Ma

**Affiliations:** Department of Histology and Embryology, College of Basic Medical Sciences, Dalian Medical University, Dalian 116044, China; hanying1113@foxmail.com (Y.H.); chenle182@163.com (L.C.); ljy8787@163.com (J.L.); chenjie1355@yeah.net (J.C.); 15797037600@163.com (C.W.); guoyu0710@163.com (Y.G.); 13889401757@163.com (X.Y.); zhangchenghong9@163.com (C.Z.); hychu2013@163.com (H.C.)

**Keywords:** Alzheimer’s disease, HDAC inhibitor, β-amyloid, synapse, AMPA receptor

## Abstract

As a neurodegenerative disease, Alzheimer’s disease (AD) seriously affects the health of older people. Changes in synapses occur first over the course of the disease, perhaps even before the formation of Aβ plaques. Histone deacetylase (HDAC) mediates the damage of Aβ oligomers to dendritic spines. Therefore, we examined the relationship between HDAC activity and synaptic defects using an HDAC inhibitor (HDACI), BG45, in the human neuroblastoma SH-SY5Y cell line with stable overexpression of Swedish mutant APP (APPsw) and in APP/PS1 transgenic mice during this study. The cells were treated with 15 μM BG45 and the APP/PS1 mice were treated with 30 mg/kg BG45. We detected the levels of synapse-related proteins, HDACs, tau phosphorylation, and amino-3-hydroxy-5-methyl-4-isoxazolepropionic acid (AMPA) receptors using Western blotting and immunohistochemistry. We also measured the expression of cytoskeletal proteins in the cell model. The mRNA levels of the glutamate ion receptor alginate subunit 2 (GRIK2), sodium voltage-gated channel beta subunit (SCN3B), synaptophysin (SYP), Grm2 (the gene encoding glutamate receptor subunit 2 (GluR2)), Grid2IP, glutamate receptor interacting protein 1 (GRIP1), and GRIP2 were detected to explore the effects of the HDACI on regulating the expression of synaptic proteins and AMPA receptors. According to our studies, the expressions of HDAC1, HDAC2, and HDAC3 were increased, which were accompanied by the downregulation of the synapse-related proteins SYP, postsynaptic dendritic protein (PSD-95), and spinophilin as early as 24 h after transfection with the APPsw gene. BG45 upregulated the expression of synapse-related proteins and repaired cytoskeletal damage. In vivo, BG45 alleviated the apoptosis-mediated loss of hippocampal neurons, upregulated synapse-related proteins, reduced Aβ deposition and phosphorylation of tau, and increased the levels of the synapse-related genes GRIK2, SCN3B, SYP, Grm2, and Grid2IP. BG45 increased the expression of the AMPA receptor subunits GluA1, GluA2, and GluA3 on APPsw-transfected cells and increased GRIP1 and GRIP2 expression and AMPA receptor phosphorylation in vivo. Based on these results, HDACs are involved in the early process of synaptic defects in AD models, and BG45 may rescue synaptic damage and the loss of hippocampal neurons by specifically inhibiting HDAC1, HDAC2, and HDAC3, thereby modulating AMPA receptor transduction, increasing synapse-related gene expression, and finally enhancing the function of excitatory synapses. BG45 may be considered a potential drug for the treatment of early AD in further studies.

## 1. Introduction

Alzheimer’s disease (AD) is a degenerative neurological disease characterized by progressive cognitive dysfunction that is sufficient to disrupt daily life. With the increasing prevalence of AD, the need for early diagnosis and treatment is even more urgent. The pathogenesis of AD is complex and diverse, and the etiology has not yet been fully clarified. The pathogenesis of AD is closely related to a variety of factors, including genetic, immune, and environmental factors [1]. For many years, research has focused on the β-amyloid (Aβ) cascade hypothesis, suggesting that AD pathogenesis starts with the production and abnormal cleavage of amyloid precursor protein (APP) by β- and γ-secretase [2]. When the Aβ monomer is overproduced, it easily misfolds and is converted into dimers or polymers after structural transformation [2]. This material is called an Aβ oligomer. The Aβ oligomer is the most neurotoxic form of Aβ and exists as an intermediate in the polymerization of Aβ [3]. The overproduction of Aβ will not only exacerbate the aggregation of Aβ but also lead to the formation of plaques [4]. The increase in soluble Aβ levels changes the activity of each kinase, triggers the hyperphosphorylation of the microtubule-associated tau protein, and leads to its oligomerization, which destabilizes microtubules and causes them to disintegrate into filaments; finally, after further condensation, tau forms insoluble neurofibrillary tangles (NFTs) [5]. Intracellular and extracellular Aβ induce neurotoxicity, leading to neuronal damage and death, as well as synaptic damage [6]. Dysregulation of the synaptic plasticity-related mechanisms leads to synaptic dysfunction, which generates senile plaques (SPs) and NFTs that result in abnormal synaptic communication and synaptic damage that causes further neuronal damage and death [7].

However, most of the drugs targeting Aβ, e.g., solanezumab, have failed in AD drug experimental studies [8]. Aβ appears in the brain and starts to affect it 10 to 15 years before symptoms are observed [9]. The failure of previous drug experiments might be due to the late intervention. Researchers found that an impairment in synaptic function appeared before the emergence of Aβ oligomers, and the synaptic impairment was likely to be an earlier event in individuals with AD [10]. Researchers have used optogenetics to restore the density of dendritic spines and increase synaptic plasticity to ameliorate early memory impairment in models of AD [11]. However, this method still involves invasive technology. Based on the results described above, we considered whether the identification of targeted drugs that rescue synaptic damage and neuron loss in the earlier stages of AD to effectively prevent and delay the occurrence of AD is possible.

The occurrence and development of AD may result from the interaction of multiple factors that may induce epigenetic changes, such as aging, genetic mutations, metabolic and nutritional disorders, obesity, and inflammation. Epigenetic changes that modify gene expression include DNA methylation and histone acetylation. In studies assessing the level of acetylated histones in APP/PS1 transgenic mice, researchers proposed that epigenetic mechanisms are related to the changes in synaptic function and memory associated with AD [12]. In addition, histone deacetylase (HDAC) activity is increased in an AD mouse model. HDACs mediate Aβ oligomerization to damage dendritic spines, while histone deacetylase inhibitors (HDACIs) attenuate this phenomenon [13]. HDACs regulate the chromosome structure and gene transcription together with histone acetyltransferases (HATs). HATs acetylate histones, facilitate DNA and histone depolymerization, and relax nucleosome structure, enabling transcription factors to interact with specific DNA sites to activate gene transcription; HDACs deacetylate histones and tightly bind negatively charged DNA, causing it to become supercoiled and inhibiting gene transcription. HDACs are classified into the following three types according to whether the proteins are homologous to yeast: Class I (HDAC1, 2, 3, and 8) regulates the acetylation of histones; Class II (HDAC4, 5, 6, 7, 9, and 10) regulates the acetylation of histones and non-histone proteins; and Class III is nicotinamide adenine dinucleotide (NAD)-dependent deacetylase associated with cellular aging and the regulation of energy metabolism. In the hippocampus of aged mice, HDAC2 overexpression modulates the decrease in dendritic spine density and shows a negative correlation with recognition memory [14], indicating that synaptic plasticity might also be negatively regulated by HDAC2. Furthermore, HDAC4 and HDAC5 levels were shown to be sensitive to treatment in a rodent model of AD [15,16]. A decrease in the levels of the functionally active (phosphorylated at S498) form of HDAC5 is associated with a significant improvement in the AD-like phenotype of 3xTg-AD mice [16].

A number of studies have indicated that Class I HDACIs promote gene transcription and the formation of new synapses [17]. Therefore, a study of drugs targeting members of the HDAC family may provide a new direction in AD research. Shuang-shuang Yang et al. suggested that the development of HDACIs might be a potential therapeutic direction in AD treatment [18]. Recently, Polis B et al. also considered HDACIs as promising AD-modifying agents [19]. The specific inhibition of HDAC3 has been shown to decrease the deposition of Aβ1-42 and the phosphorylation of tau protein and increase BDNF mRNA expression in the 3xTg-AD mouse model [20]. Some researchers have noted that the targeted inhibition of Class I HDACs alters or even reverses memory impairment in transgenic mice in behavioral assays using the APP/PS1 mice injected with a systemic HDACI [21].

In the present study, SH-SY5Y cells transfected with the APP Swedish mutant gene and APP/PS1 transgenic mice were used as cellular and animal models of AD and were treated with a Class I HDACI (BG45) to explore the early synaptic damage in AD models and the protective effect and mechanism of BG45 against the damage caused by AD to provide new insights into clinical interventions and treatments for AD.

## 2. Results

### 2.1. APP, Aβ, and p-tau/tau Levels Were Increased in Stably Transfected SH-SY5Y Cells

**Figure 1:**
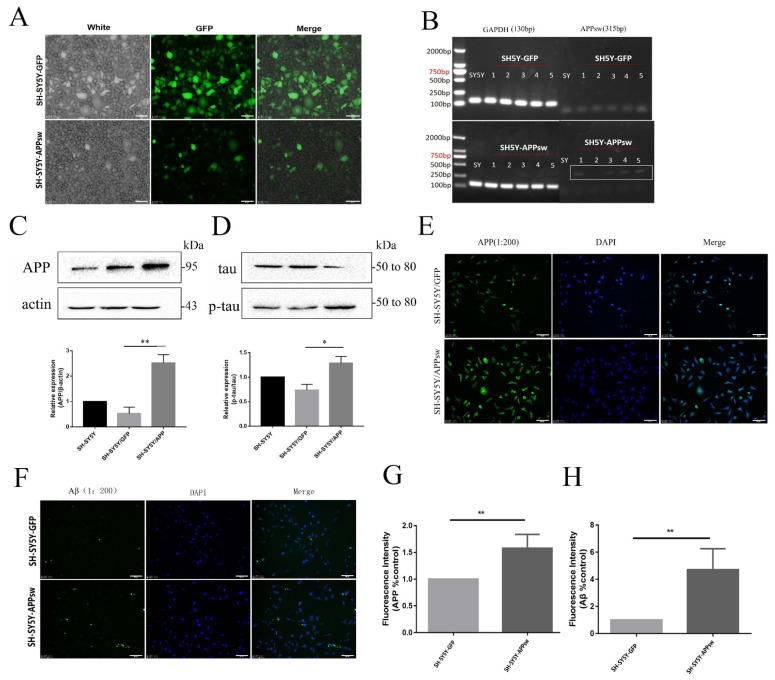
SH-SY5Y cells transfected with the APPsw gene were established as an AD cell model. (**A**) The transfection efficiency was observed in SH-SY5Y cells. (**B**) The expression of the APPsw gene was detected in different SH-SY5Y cell clones using RT-PCR. (**C**) Immunoblot analysis of APP levels in GFP- and APPsw-positive cells. Quantification of APP levels normalized to β-actin levels, and the results are presented as a % of the control and showed significant differences between SH-SY5Y cells and APPsw-positive cells (*p* < 0.01). (**D**) Immunoblot analysis of tau and p-tau levels in GFP- and APPsw-positive cells. Quantification of p-tau levels normalized to tau levels, and the results are presented as a % of control and showed significant differences between SH-SY5Y cells and APPsw-positive cells (*p* < 0.05). (**E**,**G**) Immunofluorescence staining for APP in GFP- and APPsw-positive cells (*p* < 0.05). (**F**,**H**) Immunofluorescence staining for Aβ in GFP- and APP-positive cells (*p* < 0.05). Scale bar = 50 μm (**A**,**E**,**F**). Significant differences were determined using Student’s *t*-test. All values are presented as the means ± SD from three experiments. * *p* < 0.05, ** *p* < 0.01.

SH-SY5Y cells were stably transfected with APPsw to induce APP expression and Aβ secretion and subsequently produce an AD model in vitro. CCK-8 assays were performed to determine the appropriate concentrations of puromycin. Normal SH-SY5Y cells died when the screening time was 5 days and the concentration of puromycin was 4 μg/mL. The transfection efficiency was observed under a fluorescence microscope (Figure 1A). The cells were selected based on puromycin resistance (4 μg/mL). The expression of the APPsw gene in transfected cells was detected using RT-PCR. APPsw-positive cells (SH-SY5Y cells transfected with APPsw) expressed the APPsw gene (Figure 1B). Moreover, the expression of the APP protein in both GFP-positive cells (SH-SY5Y cells transfected with *p-EGFP-N2* for the control group) and APPsw-positive cells was determined by performing Western blotting and immunofluorescence staining. The APPsw-positive cells showed a significant increase in APP expression compared to the GFP-positive cells (*p* < 0.01) (Figure 1C,D). Immunofluorescence staining was also performed to assess the levels of APP and Aβ in the GFP-positive and APPsw-positive cells. APP was expressed at significantly higher levels in APPsw-positive cells than in GFP-positive cells (Figure 1E). The APPsw-positive cells also exhibited a higher Aβ level than the GFP-positive cells (*p* < 0.01) (Figure 1F). Furthermore, because tau protein is hyperphosphorylated and abnormally accumulates in axons, dendrites, and cell bodies in subjects with AD, Western blot analysis was performed to detect the levels of tau protein; the level of tau phosphorylation was substantially increased in the APPsw-positive cells compared to GFP-positive cells (*p* < 0.05) (Figure 1D).

### 2.2. PSD-95 Was Significantly Downregulated in APPsw-Positive Cells at Different Time Points

**Figure 2:**
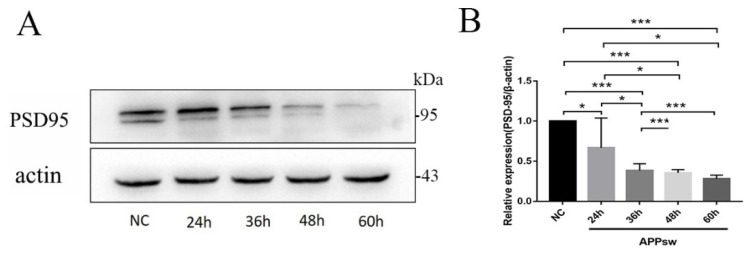
PSD-95 expression in APPsw-positive cells at different time points. (**A**) Immunoblot analysis of PSD-95 levels in SH-SY5Y cells at 60 h and in APPsw-positive cells at different time points (24 h, 36 h, 48 h, and 60 h). (**B**) Quantification of PSD-95 levels normalized to β-actin levels, and the results are presented as a % of the control and showed significant differences between SH-SY5Y cells and APPsw-positive cells at 36 h (*p* < 0.001). Significant differences were determined using repeated measures ANOVA. All values are presented as the means ± SD from three independent experiments. * *p* < 0.05, *** *p* < 0.001.

Changes in PSD-95 expression partially reflect the degree of synaptic damage. The expression of PSD-95 in APPsw-positive cells was significantly downregulated at 36 h (*p* < 0.001) (Figure 2A,B), and thus three time points (24 h, 36 h, and 48 h) were selected for subsequent experiments.

### 2.3. BG45 Increased APPsw-Positive Cell Viability and Decreased the Expression of Class I HDACs (HDAC1 and 2) in APPsw-Positive Cells

**Figure 3:**
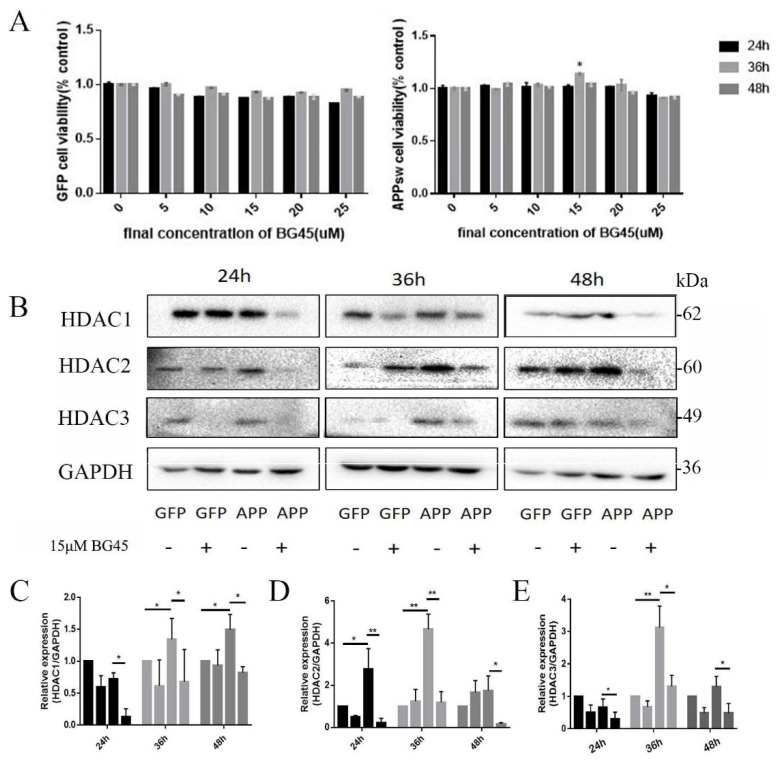
The effects of BG45 on Class I HDACs (HDAC1, 2, and 3). (**A**) The effect of BG45 on GFP-positive and APPsw-positive cell viability. (**B**) Immunoblot analysis of HDAC1, 2, and 3 levels in GFP- and APPsw-positive cells treated with vehicle or BG45 (15 μM) for different periods. (**C**) Quantification of HDAC1 levels. (**D**) Quantification of HDAC2 levels. (**E**) Quantification of HDAC3 levels. Significant differences were determined using Student’s *t*-test. All values are presented as the means ± SD from three independent experiments. * *p* < 0.05, ** *p* < 0.01.

Based on the changes in PSD-95 protein expression in APPsw-positive cells at different time points, the CCK-8 assay was used to determine the optimal time and concentration for BG45 treatment. Different concentrations of BG45 had no significant effects on GFP-positive cells at different times. Meanwhile, the viability of APPsw-positive cells treated with 15 μM BG45 for 36 h was significantly increased compared with the other groups (*p* < 0.05) (Figure 3A).

Class I HDACs (HDAC1, 2, and 3) were detected in each group at 24 h, 36 h, and 48 h to assess the expression of HDACs in APPsw-positive cells at different times and determine the effect of BG45 on HDAC expression (Figure 3B). Compared to the GFP group, the expression of HDAC1 in the APPsw group was significantly increased at 36 h and 48 h (*p* < 0.05 and *p* < 0.05) (Figure 3C). HDAC2 expression was significantly increased at 24 h and 36 h (*p* < 0.05 and *p* < 0.01) (Figure 3D). HDAC3 expression in the APPsw group was significantly increased at 36 h (*p* < 0.01) (Figure 3E). The data suggest that the levels of HDAC1, HDAC2, and HDAC3 were upregulated in the APPsw-transfected cells. Following BG45 treatment, the expression of HDAC1, HDAC2, and HDAC3 in BG45-treated APPsw-positive cells was significantly decreased at each time point compared to that in APPsw-positive cells without BG45 treatment (*p* < 0.05, *p* < 0.05, and *p* < 0.05; *p* < 0.01, *p* < 0.01, and *p* < 0.05; and *p* < 0.05, *p* < 0.05, and *p* < 0.05, respectively) (Figure 3C–E).

### 2.4. BG45 Significantly Decreased the Expression of the AD-Related Protein APP in APPsw-Positive Cells

**Figure 4:**
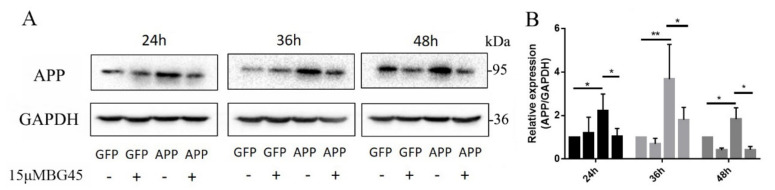
Effects of BG45 on the levels of APP. (**A**) Immunoblot analysis of APP levels in GFP- and APPsw-positive cells treated with vehicle or BG45 (15 μM). (**B**) Quantification of APP levels. Significant differences were determined using Student’s *t*-test. All values are presented as the means ± SD from three independent experiments. * *p* < 0.05, ** *p* < 0.01.

When we concluded that the expression of HDAC1 and HDAC2 was decreased by BG45 treatment of APPsw-positive cells at different time points, we further examined whether BG45 altered the expression of APP (Figure 4A). Compared with the GFP group, the expression of APP in the APPsw group was significantly increased at 24 h, 36 h, and 48 h (*p* < 0.05, *p* < 0.01, and *p* < 0.05, respectively) (Figure 4B). However, APP expression in the BG45-treated APPsw group was significantly decreased compared to that in the APPsw-positive cells without BG45 treatment (*p* < 0.05, *p* < 0.05, and *p* < 0.05, respectively) (Figure 4B).

### 2.5. BG45 Significantly Increased the Expression of SYP, PSD-95, Spinophilin, and F-Actin

**Figure 5:**
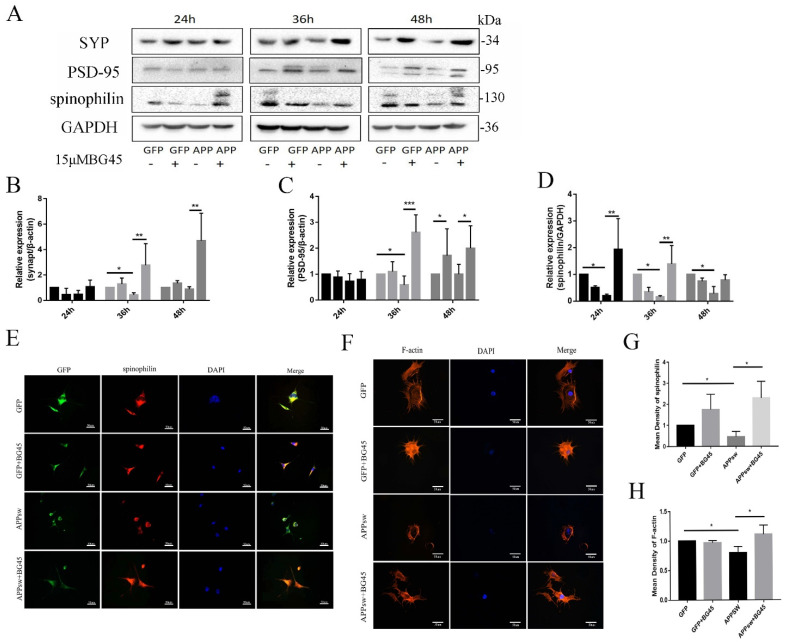
The effects of BG45 on the levels of synapse-related proteins and F-actin. (**A**) Immunoblot analysis of SYP, PSD-95, and spinophilin levels in GFP- and APPsw-positive cells treated with vehicle or BG45 (15 μM) for different periods (24 h, 36 h, and 48 h). (**B**) Quantification of SYP levels. (**C**) Quantification of PSD-95 levels. (**D**) Quantification of spinophilin levels. (**E**) Immunofluorescence staining for spinophilin in GFP- and APPsw-positive cells treated with vehicle or BG45 (15 μM) for 36 h. (**F**) Immunofluorescence staining for phalloidin/F-actin in GFP- and APPsw-positive cells treated with vehicle or BG45 (15 μM) for 36 h. (**G**) Quantification of spinophilin levels. (**H**) Quantification of F-actin levels. Scale bar = 50 μm (**E**,**F**). Significant differences were determined using Student’s *t*-test. All values are presented as the means ± SD from three independent experiments. * *p* < 0.05, ** *p* < 0.01, and *** *p* < 0.001.

Next, we investigated whether BG45 altered the expression of the synapse-related proteins SYP, PSD-95, and spinophilin (Figure 5A). For APPsw groups without BG45 treatment measured at different time points, we found that the expression of SYP and PSD-95 was significantly decreased at 36 h compared to the levels detected at 24 h (*p* < 0.05 and *p* < 0.05, respectively) (Figure 5B,C). The expression of spinophilin in the APPsw group without BG45 treatment was significantly decreased at 24 h, 36 h, and 48 h compared to that in GFP groups (*p* < 0.05, *p* < 0.05, and *p* < 0.05) (Figure 5D). However, as we predicted, BG45 increased the decrease in the expression of synapse-related proteins. After BG45 treatment for different periods, BG45-treated cells showed significant increases in the expression of SYP and PSD-95 at 36 h and 48 h compared to the APPsw group without BG45 treatment (*p* < 0.01, *p* < 0.01 and *p* < 0.001, *p* < 0.05, respectively) (Figure 5B,C); spinophilin expression was also significantly upregulated at 24 h and 36 h compared with that in the APPsw group without BG45 treatment (*p* < 0.01 and *p* < 0.01, respectively) (Figure 5D). In addition, immunofluorescence staining was conducted to analyze spinophilin morphologically. Compared with the GFP control group, the expression of spinophilin in the APPsw group was significantly decreased (*p* < 0.05), while significantly higher expression was detected in the BG45-treated APPsw group than in the APPsw group (*p* < 0.05) (Figure 5E,G).

Furthermore, F-actin staining using phalloidin revealed that the number of cell processes was decreased, the length of protrusions decreased, and the expression of F-actin was significantly decreased in the APPsw group compared with the GFP control groups (*p* < 0.05). Compared with the APPsw group, the BG45-treated group exhibited significantly repaired cytoskeletal damage (*p* < 0.05) (Figure 5F,H).

### 2.6. BG45 Increased the Cell Surface Levels of the Amino-3-hydroxy-5-methyl-4-isoxazolepropionic Acid (AMPA) Receptor Subunits GluA1 and GluA2

**Figure 6:**
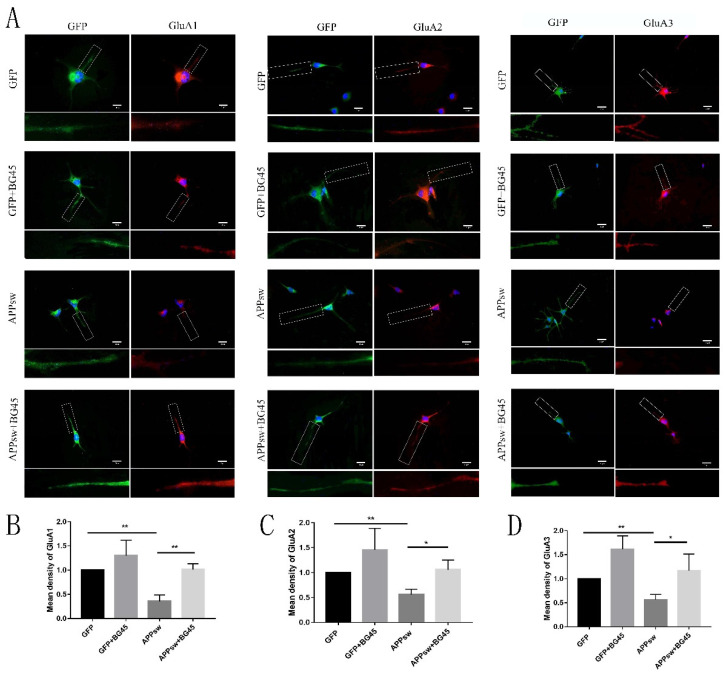
BG45 increased the cell surface levels of AMPA receptor subunits. (**A**) Immunostaining for cell surface GluA1, GluA2, and GluA3 receptors on GFP- and APPsw-positive cells treated with vehicle or BG45 (15 μM). The lower images show the positive cells magnified in the white box. (**B**) Quantification of the cell surface GluA1 levels. (**C**) Quantification of the cell surface GluA2 levels. (**D**) Quantification of the cell surface GluA3 levels. Scale bar = 50 μm (**A**). Significant differences were determined using Student’s *t*-test. All values are presented as the means ± SD from three independent experiments. * *p* < 0.05, ** *p* < 0.01.

AMPARs play a central role in modulating excitatory synaptic transmission in the central nervous system (CNS). We investigated the effect of BG45 on AMPA receptor trafficking. The detection of fluorescence on the surface of living cells (Figure 6A) showed significantly decreased expression levels of the AMPAR subunits GluA1 (*p* < 0.01), GluA2 (*p* < 0.01), and GluA3 (*p* < 0.01) (Figure 6B–D) in the APPsw-positive cells compared with those in the GFP-positive cells. BG45 significantly increased the levels of the AMPA subunits GluA1 (*p* < 0.01), GluA2 (*p* < 0.05), and GluA3 (*p* < 0.05) (Figure 6B–D) in APPsw-positive cells compared with those in APPsw groups without BG45 treatment.

### 2.7. BG45 Alleviated Neuronal Loss in the Hippocampus of APP/PS1 Transgenic Mice

**Figure 7:**
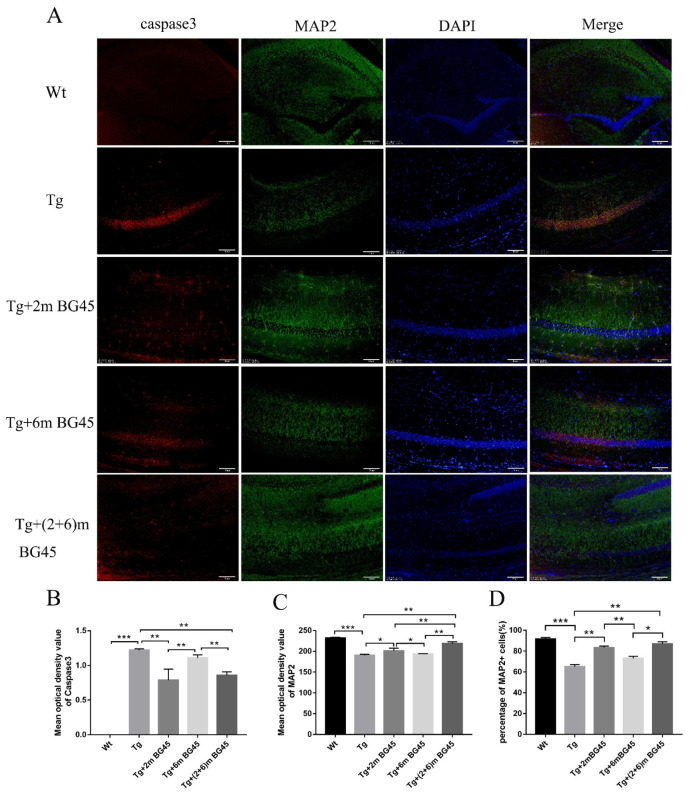
Protective effect of BG45 on hippocampal neurons in APP/PS1 transgenic mice (*n* = 5). (**A**) Immunofluorescence staining for the caspase-3 and MAP2 proteins in hippocampal neurons from animals treated with vehicle or BG45. (**B**) Quantification of the caspase-3 protein levels. (**C**) Quantification of the MAP2 protein levels. (**D**) Percentage of MAP2+ cells. Scale bar = 50 μm (**A**). Significant differences were determined using one-way ANOVA. All values are presented as the means ± SD from three independent experiments. * *p* < 0.05, ** *p* < 0.01, and *** *p* < 0.001, *n* = 5 mice per group.

Immunofluorescence staining was performed to detect the co-expression of caspase-3 and MAP2 in hippocampal neurons from APP/PS1 mice and to explore the protective effect of BG45 on hippocampal neurons in vivo. Compared with the wild-type group, the hippocampal neurons in the Tg group showed a stronger caspase-3 positive signal. Moreover, MAP2, a neuronal marker, exhibited weak co-expression. After HDACI treatment, caspase-3 expression in the Tg+2m BG45 group and the Tg+(2+6)m BG45 group decreased significantly compared with the Tg group (*p* < 0.01, *p* < 0.01); however, MAP2 expression increased significantly (*p* < 0.01, *p* < 0.05), and the level in the Tg+(2+6)mBG45 group was greater than that in the Tg+2mBG45 group (*p* < 0.01) (Figure 7A).

### 2.8. BG45 Decreased Aβ Deposition and the Level of the Phosphorylated tau Protein in the Hippocampus of APP/PS1 Transgenic Mice

**Figure 8:**
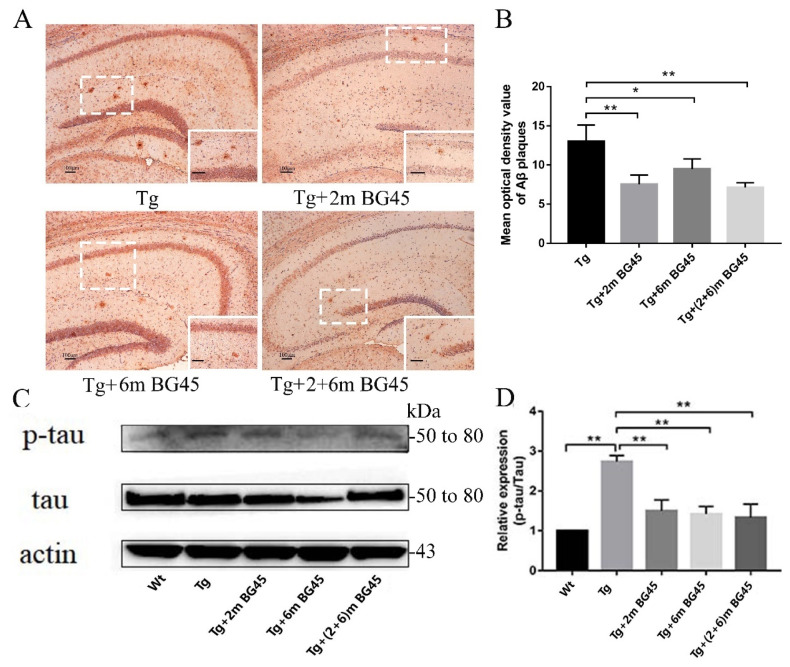
BG45 decreased Aβ deposition in hippocampal neurons and the level of the phosphorylated tau protein in APP/PS1 transgenic mice (*n* = 5). (**A**) Immunohistochemistry showing Aβ deposition in hippocampal neurons from mice treated with vehicle or BG45. The lower right corner of the image shows a magnified image that clearly depicts Aβ deposition. (**B**) Quantification of Aβ deposition. (**C**) Western blot showing p-tau/tau levels in hippocampal neurons from mice treated with vehicle or BG45. (**D**) Quantification of the level of the phosphorylated tau protein. Scale bar = 100 μm (**A**). Significant differences were determined using one-way ANOVA. All values are presented as the means ± SD from three independent experiments. * *p* < 0.05, ** *p* < 0.01, *n* = 5 mice per group.

Immunohistochemistry was used to detect Aβ deposition in the brains of mice from each experimental group. Plaques were observed in all APP/PS1 transgenic mice at 6 m, and the greatest number of plaques was observed in the Tg group. Compared with the Tg group, the plaque numbers in the HDACI treatment groups decreased significantly (*p* < 0.01, *p* < 0.05, and *p* < 0.01), among which the numbers of plaques in the Tg+2mBG45 group and Tg+(2+6)mBG45 group showed the largest decreases, but no significant differences were observed among the Tg+2mBG45, Tg+6mBG45, and Tg+(2+6)mBG45 groups (Figure 8A).

Furthermore, Western blotting was performed to detect the level of the phosphorylated tau protein. Compared with the Wt group, the level of the phosphorylated tau protein in the Tg group was increased (*p* < 0.01), but the p-tau/tau ratio in the Tg+2mBG45, Tg+6mBG45, and Tg+(2+6)mBG45 groups decreased significantly following HDACI treatment (*p* < 0.01, *p* < 0.01, and *p* < 0.01, respectively). Consistent with the results of the Aβ plaque analysis, no significant differences were observed among these three groups (Figure 8C).

### 2.9. BG45 Inhibited HDAC1 and HDAC2 Protein Expression in the Hippocampal Neurons of APP/PS1 Mice

**Figure 9:**
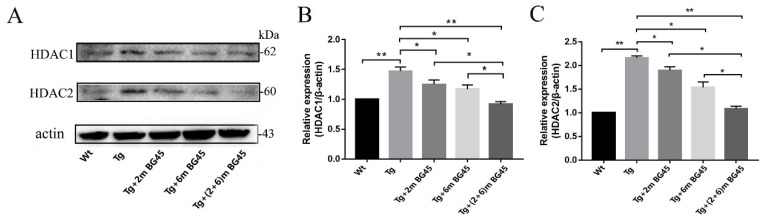
BG45 reduced HDAC1 and HDAC2 protein expression in hippocampal neurons from APP/PS1 mice (*n* = 5). (**A**) Immunoblot analysis of levels of the HDAC1 and HDAC2 proteins in the hippocampus of mice treated with vehicle or BG45. (**B**) Quantification of the HDAC1 levels. (**C**) Quantification of the HDAC2 levels. Significant differences were determined using one-way ANOVA. All values are presented as the means ± SD from three independent experiments. * *p* < 0.05, ** *p* < 0.01, *n* = 5 mice per group.

Western blotting was performed to detect the effect of BG45 on the expression of HDAC1 and HDAC2 in the hippocampus of APP/PS1 transgenic mice. HDAC1 and HDAC2 were expressed at high levels in the Tg group. Compared with the Tg group, HDAC1 expression was significantly decreased in the 2mBG45, 6mBG45, and 2+6mBG45 groups (*p* < 0.05, *p* < 0.05, and *p* < 0.01, respectively). The expression of HDAC2 in the Tg+2mBG45, Tg+6mBG45, and Tg+(2+6)mG45 groups was significantly lower than that in the Tg group (*p* < 0.05, *p* < 0.05, and *p* < 0.01, respectively). Among the HDACI treatment groups, the effect on the Tg+(2+6)mBG45 group was significantly greater than the effects on the Tg+2mBG45 and Tg+6mBG45 groups (*p* < 0.05 and *p* < 0.05, respectively) (Figure 9A).

### 2.10. BG45 Increased the Expression of Synapse-Associated Proteins in the Hippocampal Neurons of APP/PS1 Mice

**Figure 10:**
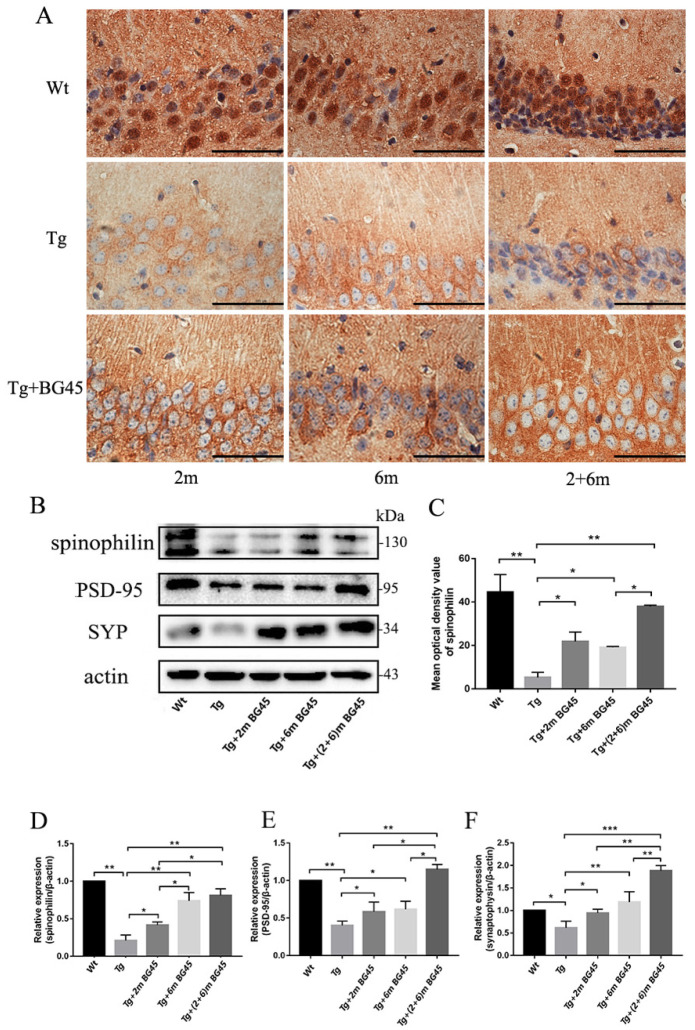
BG45 upregulates the expression of synapse-related proteins in hippocampal neurons of APP/PS1 mice (*n* = 5). (**A**) Immunohistochemical staining for the spinophilin protein in hippocampal neurons from mice treated with vehicle or BG45. (**B**) Immunoblot analyses showing levels of the spinophilin, PSD-95, and synaptophysin proteins in the hippocampus of mice treated with vehicle or BG45. (**C**) Quantification of spinophilin levels in the hippocampus. (**D**–**F**) Quantification of levels of synapse-related proteins. Scale bar = 100 μm (**A**). Significant differences were determined using one-way ANOVA. All values are presented as the means ± SD from three independent experiments. * *p* < 0.05, ** *p* < 0.01, and *** *p* < 0.001, *n* = 5 mice per group.

The effect of BG45 on the expression of spinophilin in APP/PS1 mice was detected using immunohistochemistry to evaluate the morphology of dendritic spines. As shown in Figure 10A, high spinophilin expression was observed in the hippocampus of the Wt group, while its expression in the hippocampus of the Tg group was significantly lower (*p* < 0.01). Following the administration of BG45, the spinophilin protein was expressed at higher levels in the treatment groups (Tg+2mBG45, Tg+6mBG45, and Tg+(2+6)mBG45) than in the Tg group (*p* < 0.05, *p* < 0.05, and *p* < 0.01 expression). Among the treatment groups, spinophilin was expressed at higher levels in the Tg+(2+6)mBG45 group than in the Tg+6mBG45 group (*p* < 0.05).

Subsequently, Western blotting was performed to detect the expression of synapse-related proteins. As shown in Figure 10B, compared with those in the Tg group, the expression levels of spinophilin, PSD-95, and SYP in the hippocampus of the BG45 groups were increased (*p* < 0.05, *p* < 0.01, *p* < 0.01; *p* < 0.05, *p* < 0.05, *p* < 0.01; and *p* < 0.05, *p* < 0.01, *p* < 0.001, respectively). Among the three groups, PSD-95 and SYP were expressed at significantly higher levels in the Tg+(2+6)mBG45 group than in the Tg+2mBG45 and Tg+6mBG45 groups (*p* < 0.05, *p* < 0.05; and *p* < 0.01, *p* < 0.01, respectively). Significantly higher spinophilin expression was detected in the Tg+(2+6)mBG45 group than in the Tg+2mBG45 group (*p* < 0.05). However, a significant difference was not observed compared with the Tg+6mBG45 group (*p* > 0.05).

### 2.11. Effects of BG45 on the mRNA Levels of Synapse-Related Genes in the Hippocampal Neurons of APP/PS1 Mice

**Figure 11:**
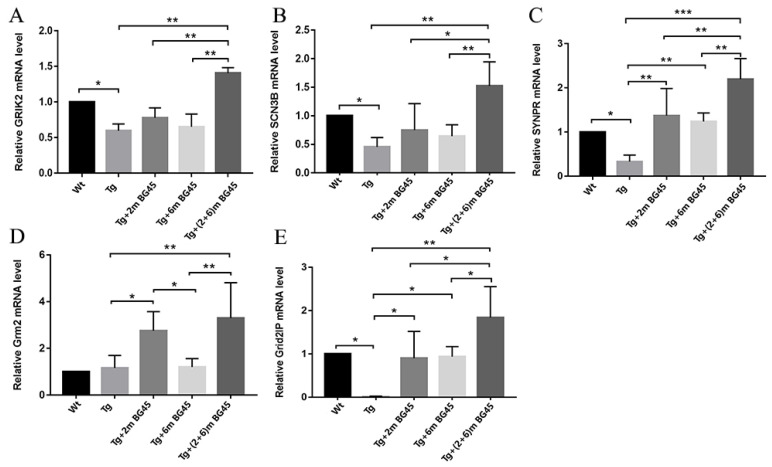
BG45 increased the levels of synapse-related genes (GRIK2, SCN3B, SYP, Grm2, and Grid2IP) in APP/PS1 mice (*n* = 5). (**A**–**E**). GRIK2, SCN3B, SYP, Grm2, and Grid2IP mRNA expression levels. Significant differences were determined using one-way ANOVA. All values are presented as the means ± SD from three independent experiments. * *p* < 0.05, ** *p* < 0.01, and *** *p* < 0.001, *n* = 5 mice per group.

Real-time PCR was used to detect the mRNA levels of synapse-related genes (glutamate ion receptor alginate subunit 2 (GRIK2), SCN3B, SYP, Grm2 (the gene encoding glutamate receptor subunit 2 (GluR2), and Grid2IP) in the hippocampus of APP/PS1 transgenic mice treated with BG45 (Figure 11A–E). The mRNA expression levels of GRIK2, SCN3B, and SYP in the Tg+2mBG45, Tg+6mBG45, and Tg+(2+6)mBG45 groups were significantly higher than those in the Tg group, especially in the Tg+(2+6)mBG45 group, which showed significantly higher mRNA levels than the Tg+2mBG45 and Tg+6mBG45 groups (*p* < 0.01, *p* < 0.01, *p* < 0.01; *p* < 0.01, *p* < 0.05, *p* < 0.01; and *p* < 0.001, *p* < 0.01, *p* < 0.01, respectively). Significantly higher mRNA expression levels of the other two genes, Grm2 and Grid2IP, were also observed in the Tg+(2+6)m BG45 group than in the other two groups (*p* < 0.01, *p* < 0.05, *p* < 0.05, respectively). 

### 2.12. Effects of BG45 on the Levels of AMPARs and Related Genes in Hippocampal Neurons of APP/PS1 Mice

**Figure 12:**
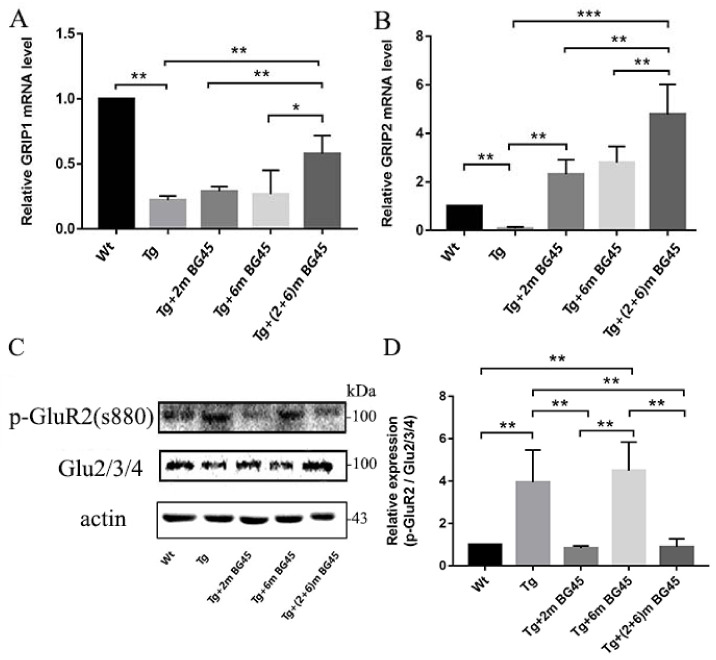
Effects of BG45 on the expression levels of glutamate receptor proteins and related genes. (**A**,**B**). GRIP1 and GRIP2 mRNA expression levels. (**C**) Immunoblot analysis of p-GluR2 (s880) and Glu2/3/4 levels in the hippocampus of mice treated with vehicle or BG45. (**D**) Quantification of p-GluR2 levels in the hippocampus. Significant differences were determined using one-way ANOVA. All values are presented as the means ± SD from three independent experiments. * *p* < 0.05, ** *p* < 0.01, and *** *p* < 0.001, *n* = 5 mice per group.

Real-time PCR was used to detect the effect of BG45 on glutamate receptor interacting protein 1 (GRIP1) and GRIP2 mRNA expression in the hippocampus of APP/PS1 transgenic mice. Compared with the Wt group, GRIP1 and GRIP2 mRNA expression levels in the Tg group were significantly decreased (*p* < 0.01 and *p* < 0.01, respectively). After BG45 treatment, their mRNA expression levels in the Tg+(2+6)mBG45 group were higher than those in the Tg+2mBG45 and Tg+6mBG45 groups (*p* < 0.01, *p* < 0.05; *p* < 0.01, *p* < 0.01, respectively) (Figure 12A,B).

Western blotting was performed to detect the expression of AMPAR subunits (Glu2/3/4) and the level of phosphorylation at the serine 880 site (S880) of GluR2. As shown in Figure 12C,D, compared with the wild-type group, the level of phosphorylation at the serine 880 site of GluR2 in the hippocampus of the Tg group was increased (*p* < 0.01). The phosphorylation level in the Tg+6mBG45 group was higher than that in the Tg+2mBG45 and Tg+(2+6)mBG45 groups (*p* < 0.01 and *p* < 0.01, respectively), but no significant difference was observed between the Tg groups (*p* > 0.05). Moreover, the phosphorylation levels in the Tg+2mBG45 and Tg+(2+6)mBG45 groups decreased significantly compared with the Tg group (*p* < 0.01 and *p* < 0.01, respectively).

## 3. Discussion

Memory deficits in patients with AD are some of the most crucial factors affecting the quality of life. Some studies have found that HDACIs targeting Class I HDACs ameliorate the contextual memory and spatial memory deficits in the mouse model of AD [21,22]. The use of phenylbutyric acid alleviates the cognitive deficit and increases the level of H4 acetylation and the expression of synapse-related proteins such as GluR1, PSD95, and MAP2 in Tg2576 mice [23]. Based on these results, drugs targeting Class I HDACs might be a promising approach to treat AD. APP is a protein that has been widely studied in the field of AD pathology [24,25]. APPsw-transfected neuron-like cells exhibit the robust expression of APP and are a reliable system that produces Aβ in vitro and models the pathological characteristics of AD [26]. SH-SY5Y cells were derived from human neuroblastoma and are induced to differentiate into neurons by adding all-trans retinoic acid (RA). In the present study, the cells were transfected with a recombinant plasmid *p-EGFP-N2-APPsw* to establish a cell model of AD. The increased expression of APP and Aβ, and the increased level of phosphorylated tau indicated that the cell model was successfully constructed and could be used in subsequent in vitro experiments. Furthermore, the APP/PS1 transgenic mouse was used as an animal AD model in vivo. We investigated the protective effects of BG45 on APPsw gene-transfected SH-SY5Y cells and APP/PS1 transgenic mice. In our previous studies, we found that BG45 ameliorated the decrease in synaptic protein expression caused by peripheral administration of exogenous Aβ in cells and animal models [27]. Therefore, in this study, we used gene transfection to induce Aβ production by the cells themselves to study the effects of an HDACI on early AD and the relationship between the HDACI, synaptic protein, synapse-related genes and receptors, and to further explore the underlying mechanism by which the HDACI increases synaptic plasticity.

The expression of HDAC2 is significantly increased in the brains of patients with AD and the CK-p25 and 5xFAD transgenic mouse AD models and AD-related neurotoxicity injury models in vitro [28]. Overexpression of HDAC2 decreases the dendritic spine density, synapse number, synaptic plasticity and memory formation, whereas overexpression of HDAC1 does not produce similar effects [29]. In addition, lentivirus-mediated overexpression of HDAC3 in the APP/PS1 mice also activates microglia cells, increases the level of Aβ and decreases the spine density [22]. In the present study, the data revealed higher expression of HDAC2 and HDAC3 in APPsw-positive cells than in control cells.

We detected the expression of AD-related proteins and synapse-related proteins at 24 h, 36 h, and 48 h to verify the correlation between HDACs and synapse-associated proteins in the AD cell model. The results showed a high APP protein level in the APPsw group at 24 h. Moreover, we observed that spinophilin expression was significantly decreased at 24 h; furthermore, SYP and PSD-95 levels began to decrease at 36 h, which is consistent with the increase in HDAC1, HDAC2, and HDAC3 levels. Kilgore, M., et al. reported that after the inhibition of HDAC1, 2, and 3 by RGFP963, the contextual fear conditioning test in APP/PS1 mice indicated that the inhibitors enhanced synaptogenesis and ameliorated the memory impairment [21]. Meanwhile, the administration of a lentivirus-mediated shRNA targeting HDAC3 reduced the amyloid burden and Aβ levels, and rescued spatial memory impairment in APP/PS1 mice [22]. Therefore, in the present study, we assumed that the decreased expression of synapse-related proteins was associated with an increased expression of HDACs. As a structural component of the presynaptic membrane and synaptic vesicles, SYP is a representative structural protein involved in the neurogenic budding reaction, and it is specifically distributed in the presynaptic membrane. It is mainly transported to the axon terminals after neurons are generated. The expression levels of SYP and PSD-95 have been used to assess the synaptic distribution and density [30]. We found that BG45 reduced the expression of HDAC1, HDAC2, and HDAC3 either prior to or while these synapse-associated proteins, including PSD-95, SYP, and spinophilin, began to change significantly in response to the increased APP levels in APPsw-positive cells.

The number of and morphological changes in dendritic spines on hippocampal neurons are considered the cellular basis of learning and memory [31]. Spinophilin is a multifunctional protein located on the dendritic spine, and it regulates the membrane and cytoskeleton and plays an important role in the central nervous system. It is closely related to the number and morphology of dendritic spines and the formation of synapses [32,33]. Our study indicated that BG45 significantly increased the expression of spinophilin. Staining for the cytoskeletal protein F-actin revealed that neurites were shorter in the APPsw group than in the control group, and the expression of F-actin was also significantly decreased. However, after treatment with 15 μM BG45, the neurites were more abundant and longer, and the cytoskeletal damage was repaired. Therefore, we speculate that in the early stage of AD, both synapses and the cytoskeleton are damaged. However, BG45 specifically alleviated synaptic damage by downregulating the expression of HDAC1, HDAC2, and HDAC3; meanwhile, it played a role in enhancing synaptic plasticity.

Nucleation-dependent polymerization occurs during Aβ deposition. When an Aβ oligomer is produced, it triggers the first nucleation step of Aβ deposition and accelerates deposition. Aβ fibrils play an important role in the later stage of Aβ aggregation, and the role of the Aβ oligomer gradually decreases [4,34]. Soluble Aβ was only detected in APP/PS1 transgenic mice at 2.5 and 3.5 months of age [35], while senile plaques were detected in the hippocampus of 7-month-old APP/PS1 mice, as shown by the specific emission of broad-spectrum blue violet excitation light from amyloid deposits [36].

Therefore, in the present study, 2 and 6 months of age were selected as the time window of administration. In addition, a group that received a double dose (one dose at 2 months and one dose at 6 months of age) was established to probe the effects of early and repeated administration. Among the treatment groups, the expression of caspase-3 and MAP2 in the hippocampus of the 6mBG45 group was significantly higher than that in the Tg+(2+6)m BG45 group or even the Tg+2m BG45 group, whereas MAP2 was expressed at lower levels in the Tg+6m BG45 group. This result indicated that the neuroprotective effect of administration at 2 months of age was more pronounced than that of administration at 6 months of age, namely, BG45 might exert an effect on early damage in this AD model. Furthermore, observations of the production of Aβ plaques and the phosphorylation of the tau protein confirmed that the Tg+2m BG45 group and the Tg+(2+6)m BG45 group showed similar changes that might be greater than those observed in the Tg+6m BG45 group. Studies with a prolonged experimental period should be conducted to further determine the long-term effects of BG45 on these parameters.

We examined the mechanism by which BG45 protects hippocampal neurons to verify the results of the in vitro experiments. First, BG45 noticeably inhibited the expression of HDAC2, especially in the Tg+(2+6)m BG45 group. Furthermore, higher expression of synaptophysin was detected in the Tg+(2+6)m BG45 group than in the Tg+2m BG45 and Tg+6m BG45 groups. In the Tg+6m BG45 group, the expression of spinophilin and PSD-95 was significantly higher than that in the other two groups, while the effect of treatment on the decreased spinophilin expression in the Tg+(2+6)m BG45 group was greater than that in the Tg+2m BG45 group. Overall, BG45 ameliorated the early damage to dendritic spines and changes in synapse-related proteins in APP/PS1 transgenic mice.

HDAC, a key enzyme involved in histone deacetylation, is mainly located in the nucleus. Knockout of the HDAC2 gene increases the expression levels of other synapse-related genes, such as GRIK2, SYP, and SCN3B [37]. Additionally, some studies used gene chips to identify genetic pathways related to synaptic function that may be activated by HDACIs [17]. In the present study, after treatment with HDACIs, the mRNA levels of GRIK2, SCN3B, SYP, Grm2, and Grid2IP, most of which are related to the expression of class I HDAC inhibitors, increased significantly compared with those in the Tg group. Therefore, among the possible gene pathways activated by HDACIs, BG45 upregulated the expression of GRIK2, SYP, SCN3B, and Grm2 and Grid2IP by inhibiting the deacetylation of HDAC2. Most of these genes are related to glutamate receptors, especially Grm2, the gene encoding GluR2. Among the treatment groups, the mRNA levels of these genes in the Tg+(2+6) mBG45 group were significantly higher than those in the Tg+2m BG45 and Tg+6m BG45 groups. We concluded that BG45 increased the expression of synapse-related genes in the early stage of AD, and the most obvious change was detected in the Tg+(2+6)m BG45 group.

Due to the changes in the expression of synapse-related genes, we focused on the AMPARs that are closely related to synaptic plasticity. AMPARs are ion channel receptors that are composed of four different GluRs: 1, 2, 3, and 4. These subunits participate in regulating neurotransmitter release, induce and maintain long-term potentiation (LTP) and long-term depression (LTD) events, and finally regulate the activities of learning and memory. Studies have shown that naturally secreted amyloid oligomers inhibit LTP in the hippocampus in vivo [10]. The absolute magnitude of LTP and LTD is usually compared to assess the deficiency in synaptic plasticity associated with AD [38]. Some researchers found that in 1-month-old transgenic mice, the induction threshold of LTP/LTD showed a tendency to increase LTP at the cost of LTD, while this phenotype was reversed to promote LTD and reduce LTP in 6-month-old transgenic mice [39]. The induction of LTP/LTD in adult AD mice is altered primarily through the synaptic recruitment and phosphorylation of AMPARs, thereby regulating developmental synapse plasticity. During LTP in the hippocampus, GluA1 is first recruited to synapses, and then GluA2 is also recruited to replace GluA1. GluA2/3 interacts with GRIP1/2 and PICK1 through its PDZ domain to form large complexes involved in AMPAR transport [40]. GRIP1 binds to GRASP, which inhibits the targeting and membrane processes of AMPARs, thus affecting synaptic plasticity.

The impaired function of AMPARs is associated with early cognitive impairment in individuals with AD. The levels of GluR1, 2, and 3 are reduced in the hippocampus of patients with AD [41], leading to a decrease in the number of dendritic spines and a loss of NMDA receptors [42,43]. Related studies have shown that the HDACI-mediated improvement in synaptic function may be associated with changes in AMPAR expression [43]. In recent years, a study confirmed the adverse effect of Aβ on AMPARs. In hippocampal neurons, Aβ1-42 oligomers reduce the expression of AMPARs on the postsynaptic membrane and reduced the membrane insertion of new AMPARs and the transport and transfer of mitochondria to dendritic spines [44]. Some researchers also found that the Aβ oligomer preferentially affected the AMPARs containing GluR2, resulting in the loss of AMPARs and dendritic spines on the surface [45]. Moreover, Aβ increased the level of GluR2 phosphorylated at serine 880 (S880) and remarkably reduced the number of GluR2 receptors [46].

Based on these findings, we first detected the expression of the AMPAR subunits in an AD cell model. The levels of GluR1, GluR2, and GluR3 were decreased in the APPsw-transfected cell membrane, while BG45 increased the expression of these receptor subunits. In the experiments using APP/PS1 transgenic mice, we found that BG45 increased the expression of GluR2/3/4 receptors in transgenic mice and reduced the level of GluR2 phosphorylated at the serine 880 site (S880). In addition, the measurement of mRNA levels in APP/PS1 transgenic mice showed that the HDACI BG45 blocked the effect of HDACs, activated gene transcription, and upregulated the expression levels of the related genes (GRIK2, SCN3B, SYP, Grm2, and Grid2IP); moreover, BG45 prevented the decrease in GluR2 expression in the AD model and reduced the level of phosphorylated GluR2 (S880 site). Therefore, the increased expression of GRIP1/2 increased the binding of GluR2 to form more transporters, which increased the transport of AMPARs; as well, the inhibitory effect of GluR2 phosphorylation at the S880 site on the GluR2 and GRIP1/2 interaction was also alleviated by the BG45-mediated decrease in GluR2 phosphorylation. Therefore, we suggest that BG45 may enhance synaptic plasticity by regulating AMPARs and altering the expression of synapse-associated proteins.

In our opinion, the neurotoxicity induced by misfolded Aβ probably leads to alterations in the molecular structure of glutamatergic receptors and causes synapse loss. The change results in reduced synaptic plasticity. In addition, HDAC2 overexpression also results in chromatin compaction and curling, inhibits gene transcription, and aggravates the damage to synaptic function. These processes all inhibit the occurrence of LTP, thereby causing impaired memory function. BG45 reduced the expression of the APP protein by specifically inhibiting Class I HDACs (HDAC1, HDAC2, and HDAC3). Additionally, by decreasing tau phosphorylation, upregulating pre- and postsynaptic protein expression, and repairing cytoskeletal damage, BG45 may have increased synaptic plasticity in cell and animal models of early AD. The underlying mechanisms might be associated with the upregulation of AMPARs and synapse-related genes, which further increased the expression of related proteins. This study provides a new insight into drug treatment for AD. Nevertheless, neurobehavioral tests are needed to show the attenuation of AD-related learning and memory deficits after BG45 treatment. Therefore, in subsequent studies, we will focus on assessing functional synaptic plasticity in cells and model animals. Moreover, gene chips could be used to identify genetic pathways related to synaptic function that may be activated by HDACIs.

## 4. Materials and Methods

### 4.1. Cell Culture, Differentiation, and Transfection

The human neuroblastoma SH-SY5Y cell line was cultured in DMEM (Gibco, Thermo Fisher Scientific, Waltham, MA, USA) supplemented with 10% fetal bovine serum (FBS) (BI, Herzliya, Israel), and 1% penicillin/streptomycin in a humidified atmosphere containing 5% CO_2_ at 37 °C. Cell differentiation was induced by treatment with 10 μM all-trans retinoic acid (RA) for 7 days. SH-SY5Y cells were transfected with a recombinant plasmid *p-EGFP-N2-APPsw* (APPsw) to establish a cell model of AD. SH-SY5Y cells transfected with the empty plasmid *p-EGFP-N2* (GFP) served as the control group. The cells were transfected using Effectene Transfection Reagent (QIAGEN, Hilden, Germany), and the cells were subsequently selected based on puromycin resistance.

### 4.2. Drug Preparation

BG45, a Class I HDAC, easily dissolves in dimethyl sulfoxide (DMSO). BG45 was first prepared into a 1 mg/mL stock solution in DMSO and then diluted 1:1000 with aqueous solution (normal saline) for use.

### 4.3. CCK-8 Assay

Cell viability was measured in 96-well plates with CCK-8 assays. Three time points (24 h, 36 h, and 48 h) and six concentrations (0 μM, 5 μM, 10 μM, 15 μM, 20 μM, and 25 μM) were established in the wells of the plate, and two replicate wells were established for each condition for detection. After cells were treated with BG45 or the vehicle for the indicated amount of time, 100 µL of CCK-8 solution were added to the medium, and then cells were incubated at 37 °C for 1 h. The absorbance was measured at 450 nm using a microplate reader (Thermo Scientific, Waltham, MA, USA). The cell viability was estimated by calculating the ratio of the optical density (OD) of the treated group to the control group.

### 4.4. Animals and Treatment

APP/PS1 transgenic mice were provided by the Nanjing Biomedical Research Institute of Nanjing University. All procedures were approved by the Institutional Animal Care and Use Committee of the Dalian Medical University in Dalian, China. Mice were housed in groups of 5 per cage under standard conditions in a temperature- and humidity-controlled facility with a 12-h light–dark cycle. Twenty male APP/PS1 transgenic mice were randomly divided into the transgenic group (Tg group) and the BG45-treated groups. Mice were divided into several groups to assess the effect of an early BG45 intervention on AD. The BG45-treated groups included a group with one administration cycle at 2 months of age (Tg+2m BG45 group), a group with one administration cycle at 6 months of age (Tg+6m BG45 group), and a group with two administration cycles at 2 months of age and 6 months of age (Tg+(2+6) mBG45 group). An intraperitoneal injection (30 mg/kg BG45, 0.2 mL each mouse) was administered for 12 consecutive days in one administration cycle. DMSO diluted with normal saline at the same concentration was used as a vehicle in the Tg group. Wild-type mice were used as a control group (Wt group). Five mice were included in each group. After all mice completed the indicated treatment, they were euthanized by cervical dislocation and the hippocampus was harvested from each mouse.

### 4.5. Western Blot Assay

After cells were lysed on ice, the samples were centrifuged at 12,000× *g* for 15 min at 4 °C. A BCA kit (Beyotime Biotechnology, Shanghai, China) was used to determine the total protein concentration of each sample. Lysates containing equivalent amounts of protein (30 µg) were separated on 10% SDS-PAGE gels and transferred to PVDF membranes (Millipore, Darmstadt, Germany ). Seven Color Prestained Protein MarkerII (SEVEN BIOTECH, Beijing, China, SW175) was used to sign the position of the proteins of interest and observe the completion of protein transfer directly. The membranes were blocked with 5% nonfat milk in Tris buffer at room temperature for 2 h. Western blotting was performed by incubating the membrane overnight at 4 °C with the following antibodies: rabbit anti-APP (1:1000, Abcam, Cambridge, UK, ab32136), rabbit anti-postsynaptic density protein 95 (PSD-95, 1:1000, Abcam, Cambridge, UK, ab18258), rabbit anti-synaptophysin (SYP, 1:1000, Abcam, Cambridge, UK, ab32127), rabbit anti-spinophilin (1:1000, Cell Signaling Technology, Boston, USA, 14136), rabbit anti-tau (1:1000, Cell Signaling Technology, Boston, MA, USA, 46687), rabbit anti-p-tau (1:1000, Cell Signaling Technology, Boston, USA, 20194), rabbit anti-HDAC1, rabbit anti-HDAC2, rabbit anti-HDAC3 (1:1000, Cell Signaling Technology, Boston, USA, 65816, Kit), rabbit anti-p-GluR2 (Ser880) (1:1000, Abbkine, Wuhan, China, ABP54637), rabbit anti-GluA2/3/4 (1:1000, Cell Signaling Technology, Boston, MA, USA, 2460S), mouse anti-GAPDH (1:5000, Proteintech, Wuhan, China, 60004-1), and rabbit anti-β-actin (1:1000, ABclonal, Wuhan, China, AC026). After three washes with 1× TBST, the membranes were incubated with a secondary antibody at room temperature for 1 h and then washed again as described above. Chemiluminescence was used to detect the proteins with the Amersham ECL Western Blotting Detection Kit (GE Healthcare Life Sciences, Pittsburgh, PA, USA). Then, the image membrane was exposed and detected with a ChemiDOC^TM^ XRS+ instrument and analyzed with Image Lab^TM^ software (BIO-RAD Laboratories, Inc., Hercules, CA, USA). The optical density of the protein band in each sample was compared with its GAPDH/β-actin band (Supplementary Materials).

### 4.6. Immunohistochemistry

The cells were seeded on 12-well slides (Solarbio, Beijing, China). Then, the cells in each well were washed with PBS, fixed with 4% PFA at room temperature for 20 min, washed three times with PBS, and permeabilized in a 0.5% Triton X-100 solution for 10 min. Mouse brains were fixed with paraformaldehyde, embedded in paraffin, and cut into sections that were 10 μm thick. After the sections were deparaffinized and completely hydrated, sodium citrate buffer was used for antigen retrieval. The cultured cells and mouse brain sections were treated as described below. After washing with PBS, nonspecific antibody binding sites were blocked by incubation with 5% BSA at room temperature for 1 h. The samples were then incubated with one of the following primary antibodies overnight at 4 ℃: rabbit anti-NeuN (1:200, Abcam, Cambridge, UK, ab177487), rabbit anti-APP (1:200, Abcam, Cambridge, UK, ab32136), rabbit anti-Aβ (1:200, Cell Signaling Technology, Boston, USA, 14975), rabbit anti-spinophilin (1:200, Cell Signaling Technology, Boston, USA, 14136), rabbit anti-caspase3 (1:200, Wanleibio, Shenyang, China, WL01992a), or mouse anti-MAP2 (1:200, Abcam, Cambridge, UK, ab5392). After washing with PBS, the samples were incubated with one of the following secondary antibodies at room temperature for 2 h: Alexa Fluor 488- or Alexa Fluor 647-conjugated goat anti-rabbit antibody (1:300, Vector Laboratories, Burlingame, CA, USA). Next, the samples were incubated with the nuclear dye, DAPI, at room temperature for 10 min for immunofluorescence staining.

For immunohistochemistry, a goat anti-rabbit IgG-biotin antibody was added dropwise to the sections and incubated for 15 min at room temperature. After washes with PBS, horseradish peroxidase-labeled streptavidin was added and allowed to bind biotin. Finally, staining was visualized with DAB and a hematoxylin counterstain. Five random slides were selected from each group and five randomly selected visual fields in the hippocampus region from each slide were observed at high magnification (×400). The objective region of images used to capture Z-stacks and the magnification are shown. Image-Pro Plus 5.1 software was used to quantitatively analyze the mean optical density (MOD) of the regions of interest (integrated optical density (IOD)/area).

### 4.7. Labeling Cytoskeletal F-Actin

The cells were seeded in 12-well slides (Solarbio, Beijing, China). The slides were washed with PBS, fixed with a 4% PFA solution, and rinsed three times. The cells were permeabilized in 0.5% Triton X-100 solution for 5 min. After washes with PBS, the cells were labeled with fluorescein-conjugated phalloidin (1:200, Sigma-Aldrich, Darmstadt, Germany ) for 20 min at room temperature. The cells were stained with DAPI at room temperature for 10 min after washing. The cells were viewed using a fluorescence microscope (Olympus, Tokyo, Japan) and the mean optical densities were quantified using Image-Pro Plus 5.1 software.

### 4.8. Live Cell Surface Immunostaining

GFP- and APPsw-transfected cells were treated with the HDACI BG45 (15 μM) or vehicle to measure cell surface levels of GluA1, GluA2, and GluA3. After 36 h, live cells were incubated with GluA1, GluA2, or GluA3 antibodies (10 μg/mL in conditioned medium) for 10 min and then fixed with 4% PFA under non-permeabilizing conditions for 5 min. Surface-labeled GluA1, GluA2, and GluA3 were detected with Alexa Fluor 647-conjugated secondary antibodies. The cells were viewed using a fluorescence microscope (Olympus, Tokyo, Japan) and the mean optical densities were quantified using Image-Pro Plus 5.1 software (Media Cybernetics, Rockville, MD, USA).

### 4.9. Reverse Transcription PCR (RT-PCR)

The samples were homogenized with Trizol reagent. Chloroform was added to facilitate RNA extraction. After an incubation at room temperature, the mixture was centrifuged to separate it into three layers, the transparent supernatant was transferred to an EP tube, and the same volume of isopropanol was added to precipitate the RNA. Then, the samples were washed with 75% ethanol three times, the supernatant was removed, the RNA samples were dried at room temperature, and the RNA concentration and purity were measured after the precipitate was dissolved. The highest purity of RNA was obtained when A260/A280 = 1.8–2.0.

The total RNA was extracted from transfected cells using Trizol reagent (Takara, Shiga, Japan) and transcribed using a reverse transcription kit (Thermo Fisher Scientific, Waltham, MA, USA) according to the manufacturer’s instructions. Gene expression levels were analyzed relative to the level of the GAPDH gene transcript. The following primers were used for RT-PCR:

APPsw, F: 5′-TCTGAAGTGAATCTGGATGCA-3′;

R: 5′-GTTCTGCATCTGCTCAAAGA-3′;

GAPDH, F: 5′-TGTGATGGGTGTGAACCACGAGAA-3′;

R: 5′-GAGCCCTTCCACAATGCCAAAGTT-3′.

The cDNA samples were mixed with primers and Dream Taq Green PCR Master Mix (Thermo Fisher Scientific, Waltham, MA, USA) in a total volume of 50 µL. The thermal cycling conditions used in the protocol were as follows: 5 min at 94 °C followed by 35 cycles at 94 °C for 1 min, 55 °C for 1 min, 72 °C for 2.5 min, and 72 °C for 10 min. The RT-PCR products were separated on a 1% agarose gel and their sizes were as follows: APP, 315 bp and GAPDH, 130 bp.

### 4.10. Real-Time Quantitative PCR (Real-Time PCR)

Total RNA was extracted from the hippocampus using Trizol reagent (Takara, Shiga, Japan) and reverse transcribed using a reverse transcription kit (Thermo Fisher Scientific, Waltham, MA, USA) according to the manufacturer’s instructions with the following primers:

SCN3B, F: 5′-ATGTGTCCAGGGAGTTTGAGT-3′

R: 5′-TTCGGCCTTAGAGACCTTTCT-3′

SYP, F: 5′-CAGCATTGACATAGCGTTTGC-3′

R: 5′-GGTTGTTTTCGCGGTACTTGT-3′

GRIK2, F: 5′-CAGCGTCGGCTCAAACATAAG-3′

R: 5′-GGTTTCTTTACCTGGCAACCTT-3′

GAPDH, F: 5′-TGTGATGGGTGTGAACCACGAGAA-3′;

R: 5′-GAGCCCTTCCACAATGCCAAAGTT-3′.

Grid2IP, F: 5′-CGGAGCGCCTCTTAGTGTC-3′

R: 5′-GCCGAAACTCTTGTTGCCTTTAT-3′

Grm2, F: 5′-CTTTGTGCGTGCCTCACTCA-3′

R: 5′-TCATAACGGGACTTGTCGCTC-3′

GRIP1, F: 5′-CAGAGGAGCGACAGACAACAT-3′

R: 5′-GGTGGTACGTGTTATGGTGATG-3′

GRIP2, F: 5′-ATGTTGGCGGTGTCACTCAAG-3′

R: 5′-TGCTGCCCTCACGTTTGAT-3′

The mRNA samples were mixed with primers and 2× TransStart Top Green qPCR SuperMix (Transgene) in a total volume of 20 µL. The thermal cycling conditions used in the protocol were as follows: 30 s at 94 °C followed by 45 cycles at 94 °C for 5 s, 60 °C for 30 s, and the dissociation step. The calculated expression of the target gene in each sample was then divided by the average calculated expression of the housekeeping gene GAPDH, which corresponds to each sample to determine a relative expression of the target gene in each sample. All experiments were performed in duplicate.

### 4.11. Statistical Analysis

All values are presented as the means ± standard deviations (SD). One-way analysis of variance (ANOVA) was used to calculate the difference in PSD at different time points or compare the population means of the three or more groups to measure the homogeneity of variance. The differences between the control group and each BG45-treated group were assessed using Student’s *t*-test. GraphPad Prism software (GraphPad Software, San Diego, CA, USA) was used for statistical analyses. Differences were considered significant at *p* < 0.05.

## Data Availability

All data supporting this study are available in the article.

## Supplementary Materials

The following supporting information can be downloaded at: https://www.mdpi.com/article/10.3390/molecules27134160/s1, Figure S1: The blots with a ladder. References [47,48,49,50,51,52,53,54,55,56] are cited in the Supplementary Materials.

## Sample Availability

Samples of the compounds are not available from the authors.
