# Supplementary material for: A Class I HDAC Inhibitor Rescues Synaptic Damage and Neuron Loss in APP-Transfected Cells and APP/PS1 Mice through the GRIP1/AMPA Pathway
Source: Molecules. 2022 Jun 29;27(13):4160. doi: 10.3390/molecules27134160 (PMC9268711; doi:10.3390/molecules27134160)
Supplement: Supplementary file 1 [file molecules-27-04160-s001.zip › molecules-1746141-supplementary.pdf]

A supplementary figure：

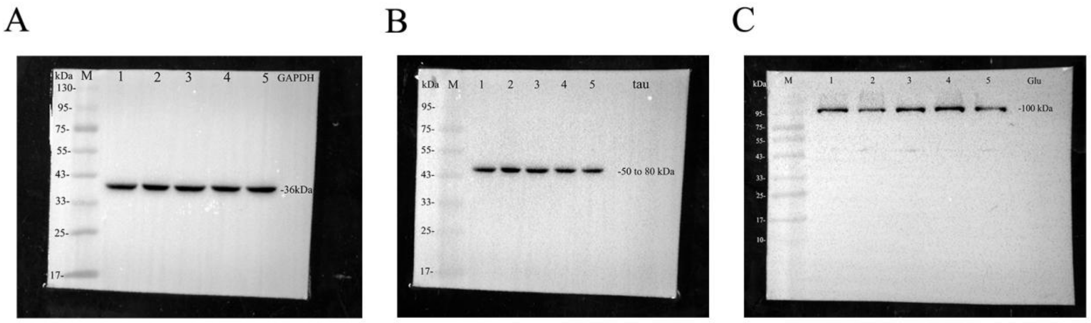

Figure S1. The blots with a ladder. Legend：The hippocampus was detected by Western blot. M: Seven Color Prestained Protein MarkerII(10-180kDa)(N0. SW175, Biotech, China), 1: WT group, 2: TG group, 3: 2m group, 4: 6m group, 5: 2+6m group. A: GAPDH(36kDa), B: tau(50-80kDa)[1], C: GluA2/3/4(100kDa)[2].

The band detected are the size that has been previously reported[1,2].

References for the other detected proteins in the manuscript: APP (95kDa)[3], postsynaptic density protein 95(PSD-95) (95kDa)[4], synaptophysin(SYP) (34kDa)[5], spinophilin (130kDa)[6], p-tau (50-80kDa)[7], p-GluR2(Ser880) (100kDa)[8], HDAC1 (62kDa), HDAC2 (60 kDa), HDAC3 (49 kDa)[9], GAPDH (36kDa)[10], β-actin (43kDa)[5].

References:

[1] Na H, Gan Q, Mcparland L, Yang JB, Yao H, Tian H, Zhang Z, Qiu WQ. Characterization of the effects of calcitonin gene-related peptide receptor antagonist for Alzheimer's disease. Neuropharmacology. 2020 May 15;168:108017.

[2] Helena Nascimento Malerba, Arthur Antonio Ruiz Pereira, Marcela Favoretto Pierrobon, Guilherme Souza Abrao, Mariana Toricelli, Eliana Hiromi Akamine, Hudson Sousa Buck, and Tania Araujo Viel. Combined Neuroprotective Strategies Blocked Neurodegeneration and Improved Brain Function in Senescence-Accelerated Mice. Front Aging Neurosci. 2021; 13: 681498.

[3] Sohrabi M, Pecoraro HL, Combs CK. Gut Inflammation Induced by Dextran Sulfate Sodium Exacerbates Amyloid-β Plaque Deposition in the AppNL-G-F Mouse Model of Alzheimer's Disease. J Alzheimers Dis. 2021;79(3):1235-1255.

[4] Huang L, Lan J, Tang J, Kang Y, Feng X, Wu L, Peng Y. L-3-n-Butylphthalide improves synaptic and dendritic spine plasticity and ameliorates neurite pathology in Alzheimer's disease mouse model and cultured hippocampal neurons. Mol Neurobiol. 2021 Mar;58(3):1260-1274.

[5] Choi GE, Lee HJ, Chae CW, Cho JH, Jung YH, Kim JS, Kim SY, Lim JR, Han HJ. BNIP3L/NIX-mediated mitophagy protects against glucocorticoid-induced synapse defects. Nat Commun. 2021 Jan 20;12(1):487.

[6] Sarrouilhe D, di Tommaso A, Métayé T, Ladeveze V. Spinophilin: from partners to functions. Biochimie. 2006 Sep;88(9):1099-113.

[7] Sun Y, Wang Y, Chen ST, Chen YJ, Shen J, Yao WB, Gao XD, Chen S. Modulation of the

Astrocyte-Neuron Lactate Shuttle System contributes to Neuroprotective action of Fibroblast Growth Factor 21. Theranostics. 2020 Jul 9;10(18):8430-8445.
[8] Xi S, Yue G, Liu Y, Wang Y, Qiu Y, Li Z, Ma P, Liu T, Jiang Y, Liang Y, Liu Q, Shi J, Chen J, Yue L. Free Wanderer Powder regulates AMPA receptor homeostasis in chronic restraint stress-induced rat model of depression with liver-depression and spleen-deficiency syndrome. Aging (Albany NY). 2020 Oct 14;12(19):19563-19584.
[9] Maiti A, Qi Q, Peng X, Yan L, Takabe K, Hait NC. Class I histone deacetylase inhibitor suppresses vasculogenic mimicry by enhancing the expression of tumor suppressor and anti-angiogenesis genes in aggressive human TNBC cells. Int J Oncol. 2019 Jul;55(1):116-130.
[10] Guan Y, Gao X, Tang Q, Huang L, Gao S, Yu S, Huang J, Li J, Zhou D, Zhang Y, Shi D, Liang D, Liu Y, Li L, Cui Y, Xu L, Chen YH. Nucleoporin 107 facilitates the nuclear export of Scn5a mRNA to regulate cardiac bioelectricity. J Cell Mol Med. 2019 Feb;23(2):1448-1457.
